# Whole exome and targeted deep sequencing identify genome-wide allelic loss and frequent *SETDB1* mutations in malignant pleural mesotheliomas

**DOI:** 10.18632/oncotarget.7032

**Published:** 2016-01-27

**Authors:** Hio Chung Kang, Hong Kwan Kim, Sharon Lee, Pedro Mendez, James Wansoo Kim, Gavitt Woodard, Jun-Hee Yoon, Kuang-Yu Jen, Li Tai Fang, Kirk Jones, David M. Jablons, Il-Jin Kim

**Affiliations:** ^1^ Thoracic Oncology Laboratory, Department of Surgery, University of California San Francisco, San Francisco, CA, USA; ^2^ Comprehensive Cancer Center, University of California San Francisco, San Francisco, CA, USA; ^3^ Department of Thoracic and Cardiovascular Surgery, Samsung Medical Center, Sungkyunkwan University School of Medicine, Seoul, Korea; ^4^ CureSeq Inc, Brisbane, CA, USA; ^5^ Department of Pathology, University of California San Francisco, San Francisco, CA, USA

**Keywords:** malignant pleural mesothelioma, multiple primary cancer, genome-wide allelic loss, exome sequencing, SETDB1

## Abstract

Malignant pleural mesothelioma (MPM), a rare malignancy with a poor prognosis, is mainly caused by exposure to asbestos or other organic fibers, but the underlying genetic mechanism is not fully understood. Genetic alterations and causes for multiple primary cancer development including MPM are unknown. We used whole exome sequencing to identify somatic mutations in a patient with MPM and two additional primary cancers who had no evidence of venous, arterial, lymphovascular, or perineural invasion indicating dissemination of a primary lung cancer to the pleura. We found that the MPM had R282W, a key *TP53* mutation, and genome-wide allelic loss or loss of heterozygosity, a distinct genomic alteration not previously described in MPM. We identified frequent inactivating *SETDB1* mutations in this patient and in 68 additional MPM patients (mutation frequency: 10%, 7/69) by targeted deep sequencing. Our observations suggest the possibility of a new genetic mechanism in the development of either MPM or multiple primary cancers. The frequent *SETDB1* inactivating mutations suggest there could be new diagnostic or therapeutic options for MPM.

## INTRODUCTION

Malignant pleural mesothelioma (MPM) is a rare malignancy with a highly unfavorable prognosis. Increased risk for MPM is strongly linked to exposure to asbestos or erionite [1–4]. Since asbestos had widely been used in different industries, the incidence of MPM in the United States is expected to steadily rise and peak over the next 20 years [1–4]. Clinical trials of single modality treatment with extrapleural pneumonectomy or pleurectomy, chemotherapy or radiation therapy have not significantly improved survival. Median survival ranges from 10–17 months [5–7].

The underlying genetic mechanisms of MPM development are not fully known. Molecular genetic analyses of MPMs have shown frequent deletions in chromosomes 1p, 3p, 4p, 4q, 6q, 9p, 13q, 14q, 15q, and 22q [8–10]. Certain tumor suppressor genes located in these regions have been implicated, such as *CDKN2A/ARF* at chromosome 9p21, *NF2* at 22q12, and *BAP1* at 3p21, [4, 11–14], but for many of these regions, the driver genes remain to be identified. Since the first report of transcriptome sequencing in MPM samples [15], large-scale genome analyses such as exome sequencing [16–18] and whole genome sequencing (WGS) [19–20] using MPM tissues or cultured cells identified potential molecular targets such as *E2F1* [17], *CUL1* [18], *MET*-related pathway genes, and *mSWI/SNF* genes, the latter of which are important for histone modification and regulation [16]. These genetic alterations suggest potentially new therapeutic targets or diagnostic markers in addition to known frequent genetic alterations (*NF2, CDKN2A*, and *BAP1*) in MPM.

Interestingly, many genes involved in histone modification and regulation mechanisms undergo germline or somatic mutations [16]. *SETDB1*, a histone methyltransferase, is reportedly a potential oncogene in lung cancer [21]. A clinical trial for the SETDB1-targeting drug mithramycin is underway for both lung cancer and mesothelioma (clinicaltrials.gov). A systematic analysis of *SETDB1* and other genes involved in histone modification could help identify genetic mechanisms for MPM. Furthermore, although previous large-scale genome studies suggested potential drug targets and diagnostic markers for MPM, most studies were confined to focal changes of mutations such as small substitutions or insertions and deletions. A systemic large genomic change such as genome-wide deletion has not been reported for MPM.

It is uncommon to observe multiple primary tumors along with MPM in the same patient. There are few, if any, suggested genetic mechanisms for the development of multiple cancers in a patient who has MPM. Here, we present a rare case of a patient who had multiple primary cancers including MPM and suggest a potentially novel genetic mechanism that explains this unusual development. Targeted deep sequencing identified a frequently mutated gene, *SETDB1*, in this patient and several others with MPM.

## RESULTS

### Clinical characteristics of an MPM patient with two additional primary cancers

**Figure 1:**
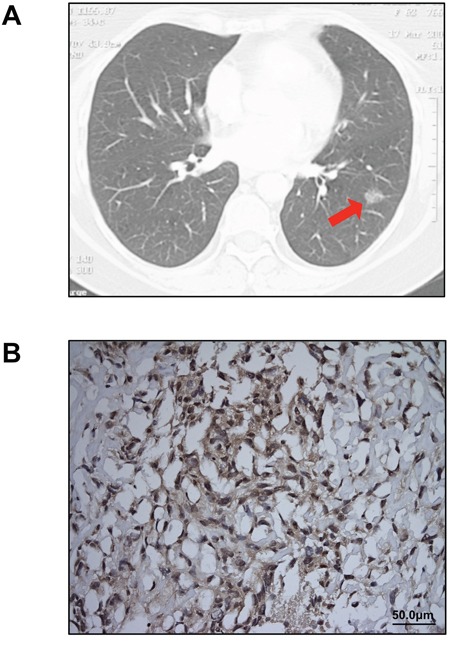
Clinical diagnosis of lung adenocarcinoma and MPM **A.** A 2 cm ground-glass opacity (arrow), later diagnosed as lung adenocarcinoma, was identified in the patient's left lower lobe on chest CT scan. No pleural thickening or implants were noted on preoperative imaging. **B.** Immunohistochemical staining for calretinin confirmed the diagnosis of MPM (original magnification 20X).

A 62-year-old Caucasian woman presented with persistent symptoms of fever, cough, chest pain and a left lower lobe consolidation on chest radiograph despite the use of antibiotics. Six months later, computed tomography of the chest revealed a discrete mass in the left lower lobe, which fine needle aspiration showed to be non-small cell lung cancer (Figure 1A). Positron emission tomography and a bone scan revealed no evidence of distant metastasis, and the patient was referred for a thoracic surgery evaluation. Her past medical history was significant for bladder cancer treated with transurethral bladder resection eight years earlier. She was a former smoker with a 30 pack-year history and had worked for a construction company while in her forties with possible, but uncertain asbestos exposure.

Five months after her initial presentation, the patient underwent a left lower lobectomy with mediastinal lymph node dissection. On thoracotomy, scattered mass lesions were incidentally discovered on the pleural surface of the diaphragm and biopsied. Final pathology revealed a T1N0M0 lung adenocarcinoma in the left lower lobe and pleural biopsies indicated sarcomatoid pleural mesothelioma, which stained positive for calretinin (Figure 1B) and was staged as T3N0M0 because there was no evidence of venous, arterial, lymphovascular, or perineural invasion. Postoperatively, two cycles of neoadjuvant chemotherapy with carboplatin and pemetrexed resulted in some disease regression. The patient had a subsequent completion left pneumonectomy. She died from mesothelioma progression four months later.

### Exome sequencing of the MPM patient's tumor revealed a genome-wide allelic loss

The development of multiple primary malignancies including MPM led us to search for underlying genetic alterations in this patient. First, we performed whole exome sequencing on the mesothelioma and adjacent normal pleural tissue as a control. Among the 11 high-confidence, non-synonymous variants identified, six were further validated by targeted deep or Sanger sequencing (Table 1). One key mutation identified was R282W in the *TP53* tumor suppressor gene. R282W is a structural mutation that renders the DNA-binding domain of p53 unstable and is a common *TP53* cancer-related mutation [22–24], but has not been described in MPM. We also identified a nonsense mutation, Y249X, of *SETDB1*, a histone methyltransferase. *SETDB1* is an oncogene frequently amplified in human lung cancers and melanomas [21, 25]. A frameshift mutation (V132fs) producing a premature stop codon in *SETDB1* was also described in the ACCMESO1 mesothelioma cell line (Cancer Cell Line Encyclopedia (CCLE) database), which suggests a potential role of *SETDB1* in MPM development.

**Table 1:** Eleven high-confidence^a^ non-synonymous variants identified by whole exome^b^ and targeted deep sequencing^c^

Gene	Chromosome	Protein change	Nucleotide change	Mutation type	QSS	Mutant ratio	Targeted sequencing
SETDB1	chr1	Y249X	747T>A	nonsense	48	0.93	validated
DYSF	chr2	R1604Q	4811G>A	missense	111	0.83	not done
ATP2C1	chr3	W460R	1378T>A	missense	99	0.86	not done
RAPGEF6	chr5	T325S^d^	973A>T	missense	51	0.85	validated
ACTB	chr7	F262L	786C>G	missense	37	0.35	validated
CASD1	chr7	V361L	1081G>T	missense	39	0.62	not done
GOT1	chr10	T326I	977C>T	missense	61	0.92	validated
GIT2	chr12	T28M	83C>T	missense	43	0.88	not done
NOD2	chr16	Y240X	720T>A	nonsense	39	0.92	validated
TP53	chr17	R282W^d^	844C>T	missense	37	0.96	validated
PSG1	chr19	E41Q	121G>C	missense	42	0.84	not done

a High-confidence is defined by quality score (QSS) greater than 30.

b Whole exome sequencing was done on SOLiD 5500 (Life Technologies) using the TargetSeq Exome Enrichment kit (Life Technologies) as previously described. [32]

c Targeted deep sequencing for validation was done on Ion Torrent PGM using a customized AmpliSeq panel (Life Technologies) as previously described. [32] (Average coverage > 2000X).

d These mutations were confirmed by Sanger sequencing.

**Figure 2:**
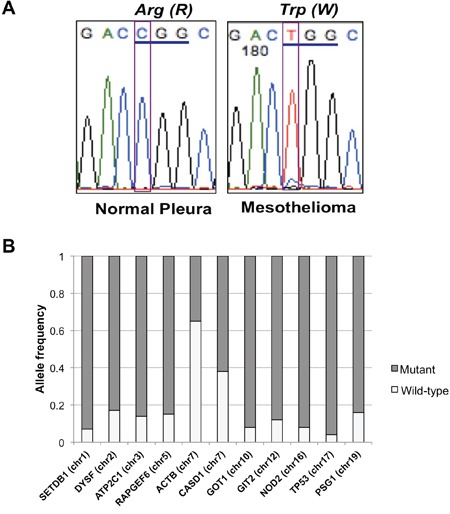
TP53 mutation and allele frequency of all variants **A.** Sanger sequencing confirmed *TP53* R282W mutation identified in exome and targeted deep sequencing of MPM. **B.** Allele frequency of variants identified in MPM with genome-wide allelic loss. Frequency of mutant alleles was higher (> 0.8) in most variants except for two located on chromosome 7.

**Figure 3:**
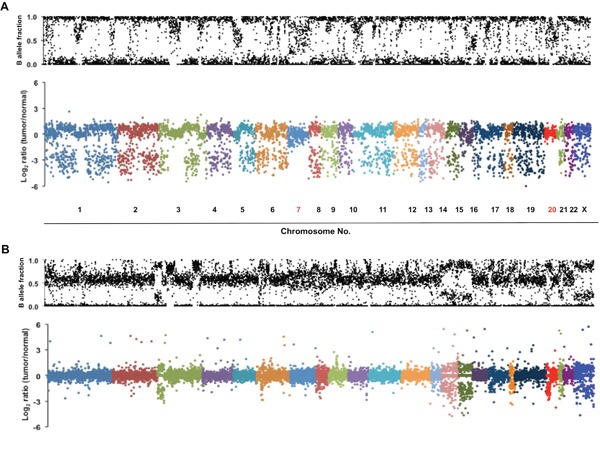
Genome-wide allelic loss identified in a MPM patient with multiple primary cancers **A.** Scatter plots show genome-wide allelic view of all variants including known SNPs identified in the patient's MPM. Allelic fraction represents ratio of wild-type to variant counts in an exome sequencing. B-allele fraction plot in the upper panel represents allelic ratio between wild-type and variant in the tumor sample. In the lower panel, the values (log2 ratio) of the x-axis are an allelic fraction of tumor normalized to that of matched normal pleura. The majority of variants on all chromosomes except chromosomes 7 and 20 show losses of either wild-type or variant allele in tumor. **B.** An example of genome-wide allelic view of tumor with frequent genetic alterations, but only with focal allelic loss. B-allele fraction plot of tumor sample in the upper panel and allelic fraction plot of tumor normalized to that of matched normal in the lower panel were newly generated from exome sequencing data previously reported [32].

Interestingly, most mutations identified in the MPM patient were highly enriched for the mutant allele, suggesting a homozygous alteration or deletion of wild-type allele when minimal contamination of normal pleura in the MPM is considered (Figure 2A). Most variants identified showed a high frequency of mutant alleles, except variants on chromosomes 7 and 20 (Figure 2B). Analyses of allelic fraction between wild-type and variants in MPM tumor (Fig 3A, upper panel), as well as tumor allelic log ratio to normal (Fig 3A, lower panel) using all variants in exome sequencing, including known SNPs, revealed that this MPM showed genome-wide allelic loss or loss of heterozygosity (LOH). However, same analyses showed a distinct pattern from another cancer that has many genetic alterations with focal allelic loss (Figure 3B). Although regional loss in chromosomes 1, 3, 4, 6, 9, 10, 13, 14, 17 and 22 has been reported in MPMs [8–10], to the best of our knowledge, this type of extensive genome-wide allelic loss has not been described in MPM.

Further genetic analysis was not possible because the patient's lung adenocarcinoma specimen was unavailable. Instead, we performed exome sequencing on a focus of atypical adenomatous hyperplasia (AAH), finding no evidence of the genome-wide allelic loss seen in MPM. However, a major driver mutation, G12C, in the *KRAS* oncogene, might have been involved in the development of this AAH. In our first targeted deep sequencing using the customized Ampliseq panel (Life Technologies) to validate mutation candidates from MPM, we sequenced six interesting candidates (*KANK4, GBP4, SETDB1, ACTB, GOT1*, and *NOD2*) in MPM. Four (*SETDB1, ACTB, GOT1*, and *NOD2*) were validated as true somatic mutations (Table 1).

### Frequent inactivating mutations of *SETDB1* in MPM

**Figure 4:**
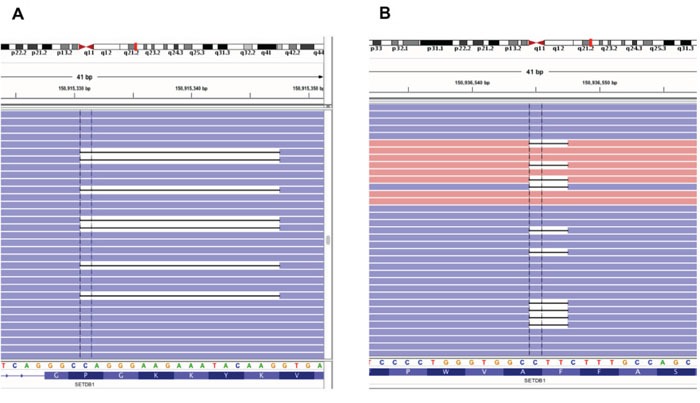
Frequent SETDB1 mutations in MPM patients Two types of deletion mutations from MPM patients, shown by an Integrative Genomics Viewer (IGV). **A.** 17 bp deletion (677_693del17) and **B.** In-frame deletion (3747_3749del) of *SETDB1* were identified from different MPM patients.

Among the mutations validated by exome and targeted sequencing in MPM tumor samples, we found that *SETDB1* had a nonsense mutation (Y249X). Because another inactivating frameshift mutation (V132fs) was reported in the mesothelioma cell line ACCMESO1, we analyzed mutations of *SETDB1* for this whole coding region by targeted deep sequencing of the whole coding exons of *SETDB1* in 77 additional primary MPM tissues, for a total sample of 78 MPM tissues from 69 MPM patients. Up to 40,000X sequencing coverage was obtained from our custom-designed targeted sequencing panel. We identified seven somatic mutations from the 69 MPM patients; these mutations have not been reported in any other databases, including 1,000 genome, COSMIC, and dbSNP. Four point mutations and three deletions were identified from a total of six MPM patients (Table 2). Among these, two samples shared the same 17 bp deletion mutation (677_693del17) (Figure 4). All these somatic mutations were validated by three independent targeted sequencings with at least 500X coverage. Ten matched normal pleural samples were available from 69 mesothelioma patients and sequenced to find any *SETDB1* variant, but none were identified. Finally, to determine whether *SETDB1* mutation was correlated with overall survival of MPM patients, we divided 69 MPM patients based on *SETDB1* mutation status. We found no significant correlation (p=0.351), probably because there were so few mutation-positive samples.

**Table 2:** *SETDB1* mutations identified in 78 MPMs from 69 patients and previously reported

ID	Protein change	^a^Nucleotide change	Mutation type	^d^PolyPhen-2	^e^SIFT	Predicted function	Mutant ratio
777T	Y249X	747T>A		nonsense		Loss of function	0.91
869T	G869E	2606G>A	missense	damaging	damaging	Damaging	0.51
163T	C911F	2732G>T	missense	damaging	tolerated	Uncertain	0.49
324T	S947C	2840C>G	missense	benign	damaging	Uncertain	0.61
970T	P226RfsX4	677_693del17	frameshift			Loss of function	0.19
981T	P226RfsX4	677_693del17	frameshift			Loss of function	0.23
1278T	F1250del	3747_3749del	in-frame del			Uncertain	0.14
^b^ACCMESO1	V132EfsX3	395_399del5	frameshift			Loss of function	
^c^NYU695	K674SfsX73	2020delA	frameshift			Loss of function	

a NM_001145415.1

b Previously reported mutation: ACCMESO1 - V132fs (CCLE, http://www.broadinstitute.org/ccle)

c Previously reported mutation: NYU695 - p.K674fs [18]

d http://genetics.bwh.harvard.edu/pph2/

e http://sift.jcvi.org/

## DISCUSSION

Our exome sequencing analysis of MPM in a patient who also had lung adenocarcinoma and a history of bladder cancer showed a rare case of MPM with genome-wide allelic loss. Although karyotyping to confirm genome-wide allelic loss would have been ideal, at the time the experiments were done, the patient had already died and no appropriate samples were available. We therefore used four different sequencing technologies (Exome, two targeted, and Sanger sequencing) and confirmed the genome-wide allelic loss.

Loss of p53 may lead tumor cells to be more vulnerable to unrepaired genomic instability in response to DNA damage or stress [22–24]. Chromothripsis involving massively rearranged chromosomal structure scattered with widespread genomic losses has been observed in several cancer genomes by analyzing their sequence fragments at the whole genome level [26–27]. Chromothripsis is strongly linked to *TP53* germline or somatic mutations [26–27]. Although we could not clearly define or classify the genome-wide allelic loss phenomenon as a chromothripsis or similar event because of the limited number of variants identified in exome sequencing analysis, we could assume that bi-allelic inactivation of the *TP53* tumor suppressor gene via R282W mutation and LOH played a critical role in the genomic abnormality observed in our MPM case. To the best of our knowledge, *TP53* R282W mutation has not been reported in MPM patients. Because *TP53* mutation is important for genomic instability such as chromothripsis, and R282W is a key mutation altering function of p53 [22-24, 26-27], it would be meaningful to further examine the relationship between *TP53* R282W and a genome-wide loss in MPM. We believe this is the first report of genome-wide allelic loss in a patient who developed multiple primary cancers including MPM. As genome-wide allelic loss can be easily overlooked by mutation-focused genetic analyses, our finding suggests that patients with multiple cancers including MPM may need to be screened for genome-wide allelic loss.

Another notable finding in this study was the high frequency of *SETDB1* mutations in MPM. Exome and WGS studies have been done in MPM or peritoneal mesothelioma samples [16–20], but none reported *SETDB1* as a new high frequent mutation in mesothelioma. We identified a somatic *SETDB1* mutation by exome sequencing, validated it by targeted deep sequencing analysis, and sequenced 77 additional MPM samples using another targeted deep sequencing panel of *SETDB1* three times, which we believe clearly rules out a technical artifact of sequencing. There are several reasons that may explain why other studies did not find that *SETDB1* was a frequent mutation in MPM, but we did. First, in previous studies, the *SETDB1* gene was screened by exome or WGS in MPM [16–20]. Some studies used a targeted sequencing panel [28–29] that did not include the *SETDB1* gene. Exome or WGS approaches have around 10-100X coverage in general, which may miss a challenging mutant allele. In contrast, our coverage range of *SETDB1* targeted deep sequencing was up to 40,000X—a difference that could explain why we detected the *SETDB1* mutation. Second, previous studies analyzed single-digit sample sizes of MPM [16–20], except for one exome analysis [18] that included 22 MPM samples. Third, different types of samples were used for mutation screening, which may have affected the detection results. We used flash-frozen tissues from MPM patients, whereas some previous studies [16–17] used cultured cells. Finally, we screened MPM samples, whereas some previous exome or WGS studies focused on peritoneal mesothelioma, another subtype [17,20].

**Figure 5:**
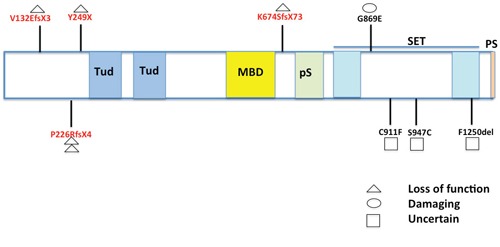
Map of SETDB1 mutations identified in MPMs Nine *SETDB1* somatic mutations identified in this study and reported previously were mapped in domains of *SETDB1*. Four of five truncating mutations were located at the N-terminal (5′) regions before Tudor (Tud) domains. All three missense and one deletion mutation were found in the SET domain. Tud: Tudor; MBD: methyl-CpG-binding domain; pS: pre-SET; PS: post-SET [30–31].

*SETDB1* has been reported to be amplified and a potential oncogene in lung cancer [21]. Increased expression of SETDB1 promotes tumor invasiveness and sensitizes anti-tumor growth by mithramycin, a SETDB1 and Sp1 inhibitor [21]. Our finding that *SETDB1* had a nonsense mutation (Y249X) and the fact that another inactivating frameshift mutation (V132fs) was reported in the mesothelioma cell line ACCMESO1, led us to hypothesize that truncating mutations such as nonsense and frameshift mutations are frequent in MPM. When we tested this using targeted deep sequencing analysis in 78 MPM samples from 69 MPM patients, we found three deletion mutations. Two were the same 17 bp deletion mutations at exon 7 and the other was an in-frame deletion mutation (Table 2) (Figure 4). The remaining three mutations were novel missense mutations. Interestingly, while all predicted loss-of-function mutations except one are located in the N-terminal, missense and in-frame deletion mutations affecting single amino acid residues are located in the SET domain of *SETDB1* (Figure 5) [30–31]. Although *SETDB1* has been suggested to have an oncogenic impact in lung cancer based on transcription level or expression data [21,25], our results suggest that at least some of the identified *SETDB1* somatic mutations are probably loss of function mutations. Functional validation and characterization should be performed to prove this, but the different mutation spectrum suggests the location of somatic mutations on the *SETDB1* functional domains may be important for oncogenic effect or tumor suppression. Exome sequencing in eight primary cultured cells from different subtypes of mesothelioma found that genes involved in histone modification and regulation mechanisms, such as *BAP1, SETD2, USP49*, and *PRMT6*, were mutated, suggesting somatic inactivation of histone modifier genes is important for mesothelioma development [16]. The same study also identified a truncating mutation of *BAP1* and whole deletion of PRMT6 [16]. These results are consistent with our finding that mutations of *SETDB1*, histone methyltransferase, are frequent in MPM, suggesting that histone modifier genes, including *SETDB1*, are potentially important in MPM. Further functional studies are required to investigate a role of *SETDB1* in MPM development.

In summary, we identified genome-wide allelic loss in a patient who had MPM and two additional primary cancers, results which suggest that careful analysis in exome sequencing is needed to detect genome-wide deletion in MPM samples with or without multiple primary cancers. The high frequency of mutations in *SETDB1* that we found suggests that this and other histone-related genes are important in MPM.

## MATERIALS AND METHODS

### DNA extraction of 78 primary MPM tissues from 69 MPM patients

DNA from a patient with multiple primary cancers (bladder, lung, and MPM), and 77 additional MPM tissues was extracted as described previously [32–33]. All samples were collected under a protocol (#11-06107) approved by the Committee for Human Research at the University of California, San Francisco. Clinical data for the 69 MPM patients is summarized in Supplementary Table 1.

### Immunohistochemistry (IHC) staining

The MPM tissue slides from the patient with multiple primary cancers were used for IHC staining with anti-calretinin antibody (# 180211, ThermoFisher) as previously described [34]. Briefly, 5μm-thick slide sections were deparaffinized with xylene and steamed in citrate for 20 minutes. They were then treated with blocking solution, washed, and incubated overnight. The slides were washed again and incubated with solutions from the Invitrogen Histostain Plus Broad Spectrum Kit (85-9643), washed again, stained with hematoxylin for 1 minute, mounted, and analyzed.

### DNA library preparation and exome sequencing

Serial samples of normal tissue, atypical adenomatous hyperplasia (AAH), and MPM tissue from the patient with multiple cancers including MPM were used for the exome sequencing library preparation. SOLiD 5500 (Life Technologies) was used for whole exome sequencing according to the manufacturer's protocol and our previous experiment [32]. Briefly, 3 ug of DNA was fragmented by Covaris S220 (Covaris), and the fragmented DNA was barcode ligated and amplified. After fragmented DNA was quantified, 500 ng of DNA was used for the TargetSeq Exome Enrichment kit (Life Technologies) to enrich the exome only. A diluted exome library was amplified by emulsion PCR and enriched. The prepared libraries were run onto the Flow Chip in SOLiD 5500.

### Targeted deep sequencing to validate somatic mutation candidates

To validate the identified mutations from the exome sequencing of the patient who had MPM and two other primary cancers, we designed a customized Ampliseq panel (Life Technologies) to sequence six mutation candidates (*KANK4, GBP4, SETDB1, ACTB, GOT1*, and *NOD2*). The Ion Ampliseq designer was used to make a targeted customized panel for these six genes according to the manufacturer's protocol. Library preparation and sequencing were also done according to our previous protocol [32].

### Targeted deep sequencing of *SETDB1* in 78 MPM tissues from 69 MPM patients

We designed another targeted panel of *SETDB1* covering all 21 coding exons spanning 6.05 kb (99.76% coverage) (CureSeq Inc, Brisbane, CA). A total of 31 amplicons were designed and sequenced in 78 primary mesothelioma tissue samples. A library was prepared according to the manufacturer's protocol (CureSeq Inc, Brisbane, CA). In brief, library preparation was performed on each sample with adaptor and barcode ligation. Sequencing adaptors and barcodes (CureSeq Inc, Brisbane, CA) for multiplex sequencing purposes were ligated to the amplicons using ligase. After purification, the library was quantified using the Agilent Bioanalyzer with High-Sensitivity DNA kits (Agilent Technologies) following the manufacturer's protocol. Library emulsion and enrichment were performed using the Ion PGM One Touch 2 and Enrichment System machine according to the manufacturer's protocol (Life Technologies). Library with Ion Sphere Particles (ISPs) was added to the reaction tube using Ion PGM Template reagents (Life Technologies). ISP emulsion and breaking were automated through the Ion PGM OneTouch 2 system. The recovered ISPs were enriched by an Ion PGM ES machine using Dynabeads MyOne Streptavidin C1 beads (Life Technologies). Sequencing was done according to the manufacturer's protocol with enriched ISPs using the 318 chips (Life Technologies) on an Ion Torrent PGM.

### Analysis of exome and targeted sequencing data

Raw sequence data were converted to fastq format using BEDtools (version 2.17.0) to perform alignment to the human reference genome (hg19, NCBI Build 37) using the Burrows_Wheeler Aligner (BWA mem, version 0.7.12). Aligned data were then grouped by sample data using picard (version 1.114). The Genome Analysis ToolKit (GATK version 3.3-0) was used to perform insertion and deletion realignment, base recalibration, and variant calling. Single nucleotide variants and insertions/deletions were detected by using GATK, and annotated afterwards with ANNOVAR [35]. Several databases were used for annotation filtering such as dbSNP and 1000 Genomes (www.1000genomes.org). All the annotated mutations were visualized by Integrative Genomics Viewer (IGV, version 2.3.43) to confirm. Python-based programs developed in-house were used to filter out common benign and recurrent artifact variants during the annotation validations with IGV. Mutation frequencies were calculated through the programs by using information obtained from processed bam files by comparing the reads containing the mutations and the total number of reads containing the region of mutations. Those samples which were validated for variants were reconfirmed through separate run process to rule out the experimental bias.

### Sanger sequencing validation

Sanger sequencing as described previously [33] was done to validate *TP53* R282W and *RAPGEF6* T325S mutations in the sample from the MPM patient who had additional primary cancers.

### Survival analysis of MPM patients according to *SETDB1* mutation status

We divided 69 MPM patients into *SETDB1* wild-type (n=62) and mutant (n=7) groups. Overall survival rates were determined using the Kaplan-Meier method in SPSS V22.0 for Mac. Survival rates were compared using the log-rank test. Two-sided P values less than 0.05 were considered statistically significant.

## SUPPLEMENTARY TABLE


