# Supplementary material for: Whole exome and targeted deep sequencing identify genome-wide allelic loss and frequent SETDB1 mutations in malignant pleural mesotheliomas
Source: Oncotarget. 2016 Jan 27;7(7):8321–31. doi: 10.18632/oncotarget.7032 (PMC4884995; doi:10.18632/oncotarget.7032)
Supplement: Supplementary file 1 [file oncotarget-07-8321-s001.pdf]

SUPPLEMENTARY TABLE

Supplementary Table S1: Summary of clinical data for 69 MPM patients

	All	*SETDB1* wild-type	*SETDB1* mutant
Number of patients	69	62	7
Mean Age	67.2	67.5	63.7
Sex, % Male	73.9%	75.8%	57.1%
Race			
Caucasian	83.6%	85%	71.4%
Latino	9.0%	6.7%	28.6%
Black	1.5%	1.7%	0%
Asian	1.5%	1.7%	0%
Other	4.5%	5.0%	0%
Smoking History			
Never smoker	42.6%	45.5%	16.7%
Former smoker	52.5%	49.1%	83.3%
Current smoker	4.9%	5.5%	0%
Mean pack years	29	30	24
Mesothelioma Exposure			
Known exposure	73.5%	71.4%	83.3%
Subtype			
Epithelioid	82.6%	82.2%	85.7%
Sarcomatoid	8.7%	8.1%	14.3%
Biphasic	8.7%	9.7%	0%
Hemithorax			
Left	34.4%	30.9%	66.7%
Right	65.6%	69.1%	33.3%
Pathologic Stage			
Stage I	45.6%	41.2%	83.3%
Stage II	5.3%	5.9%	0%
Stage III	43.9%	47.1%	16.7%
Stage IV	5.3%	5.9%	0%
Overall Survival			
Mean (months)	18.2	17.7	22.4
Median (months)	12.0	12.5	11.0
